# The Surgical Treatment of Mycetoma

**DOI:** 10.1371/journal.pntd.0004690

**Published:** 2016-06-23

**Authors:** Suleiman Hussein Suleiman, EL Sammani Wadaella, Ahmed Hassan Fahal

**Affiliations:** Mycetoma Research Centre, University of Khartoum, Khartoum, Sudan; Fundação Oswaldo Cruz, BRAZIL

## Abstract

Surgical intervention is an integral component in the diagnosis and management of mycetoma. Surgical treatment is indicated for small, localised lesions and massive lesions to reduce the mycetoma load and to enable better response to medical therapy. It is also a life-saving procedure in patients with massive disease and sepsis.

Surgical options for mycetoma treatment range from a wide local surgical excision to repetitive debridement excisions to amputation of the affected part. Adequate anaesthesia, a bloodless field, wide local excision with adequate safety margins in a suitable surgical facility, and expert surgeons are mandatory to achieve the best surgical outcome. Surgical intervention in mycetoma is associated with considerable morbidity, deformities, and disabilities, particularly in advanced disease. These complications can be reduced by educating patients to seek medical advice earlier when the lesion is small, localised, and amenable to surgery. There is no evidence for mycetoma hospital cross infection.

This communication is based on the authors’ experience in managing over 7,200 mycetoma patients treated at the Mycetoma Research Centre, University of Khartoum, Sudan.

## Introduction

**Fig 1:**
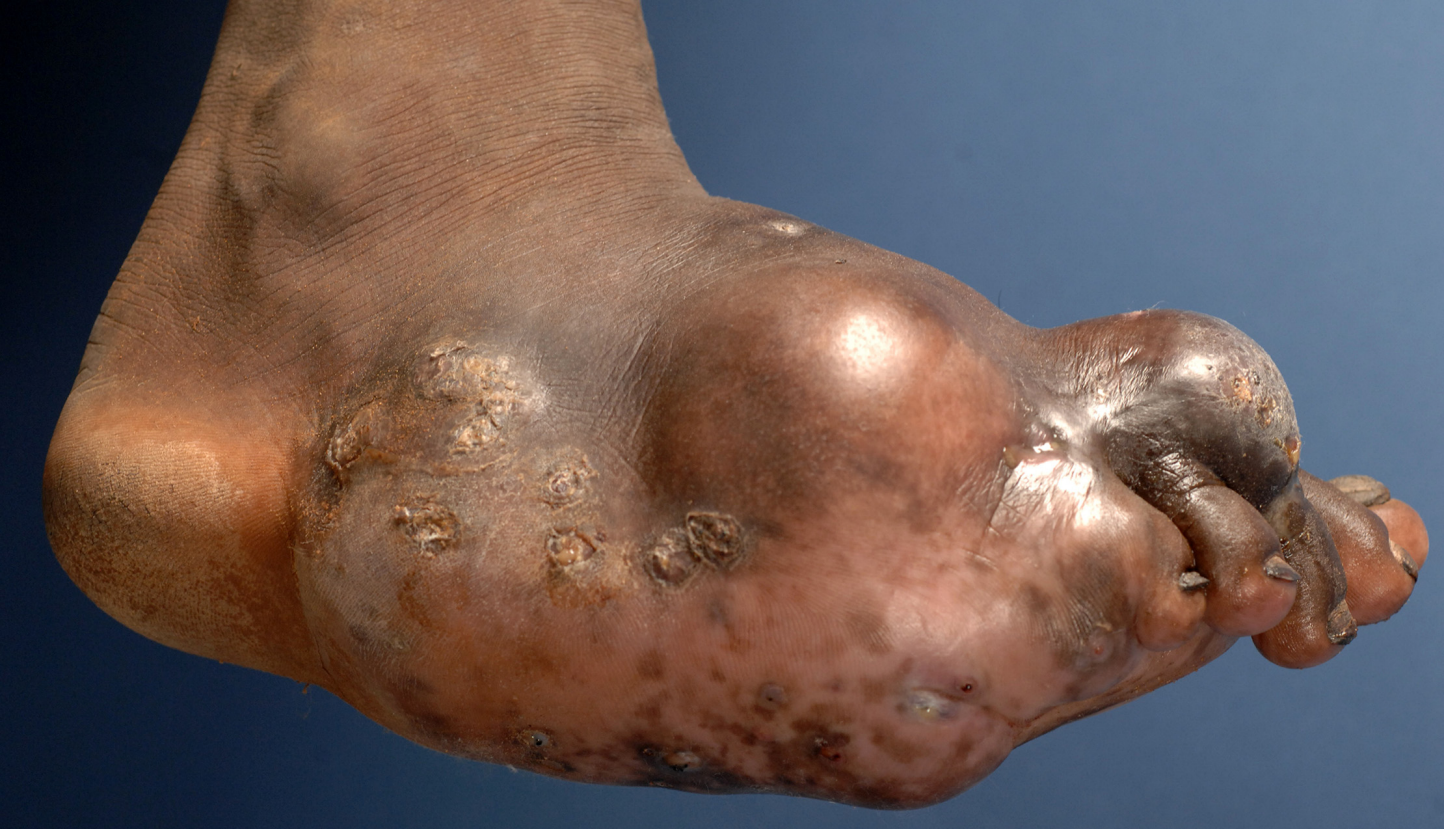
Photograph showing massive foot eumycetoma caused by *Madurella mycetomatis*.

Mycetoma is a neglected tropical disease characterised by deformity and disabilities with various medical, health, and socioeconomic impacts on the affected communities [1–3]. It is endemic in many tropical and subtropical regions, but it has been globally reported [4,5]. Mycetoma is a chronic inflammatory disease caused by true fungi or certain bacteria, hence the classifications eumycetoma and actinomycetoma, respectively. The painless subcutaneous mass, multiple sinuses formation, and purulent or seropurulent discharge frequently containing grains are the main features of mycetoma (Fig 1) [6–8]. Young adult males are commonly affected, and feet and hands are mostly involved [9–11]. Numerous tools and techniques are required to establish the diagnosis of mycetoma, and the surgical biopsy is an important tool [12–16]. To determine the extension of the lesion, several preoperative imaging techniques are required, including conventional X-ray taken in at least two views (anterio-posterior and lateral), lesion ultrasound examination, MRI, and CT scan [12,13,15].

In general, bacterial mycetoma is amenable to medical therapy with good response rates. In contrast, fungal mycetoma requires both prolonged medical therapy and surgical treatment [17–20].

## Indications for Surgery

In general, from our experience, surgical treatment for mycetoma is indicated for small, well-localised lesions in those patients who are not responding to medical therapy in order to reduce disease burden in massive lesions, allow a better response to medical therapy, and control secondary bacterial infection, and for cases in which medical therapy is contraindicated, as in pregnancy and lactation [21,22]. Surgery may be a life-saving procedure in advanced disease that is complicated by secondary bacterial infection, sepsis, massive bone involvement, and poor general condition.

Surgical biopsies are frequently performed to establish the diagnosis of mycetoma. The surgical biopsies provide tissue for histopathological examination and immunohistochemical studies [23–25]. In many centres, culture is the cornerstone of identifying the causative organisms. Grains are the source for the culture. Grains obtained by deep-seated surgical biopsy specimen are ideal, as grains discharged with pus through an open sinus are generally dead and contaminated [26,27]. Grains are also essential for the molecular identification of the causative organisms and for antigen preparation for various serological tests [28,29].

Surgical biopsies are commonly obtained by a wide local incision under ideal anaesthesia and surgical conditions. Occasionally a tru-cut needle biopsy under local anaesthesia is performed in massive lesions with wide local spread.

## Preoperative Care

Optimal surgical excision is a prerequisite for the best treatment outcome. Clinical observations suggest that incomplete surgical excision performed under local anaesthesia by an inexperienced surgeon in a rural area with poor surgical facilities is an important factor for determining disease recurrence. Prophylactic antibiotics are essential and need to be administered at induction of anaesthesia. In massive eumycetoma lesions, antifungal agents are given for period of time ranging from six to nine months to induce adequate fibrous capsule formation around the lesions, thus facilitating surgical dissection and excision (Table 1) [19].

**Table 1:** Mycetoma treatment guidelines.

Small lesions (<5 cm) without bone involvement	Wide local excision	Itraconazole 400 mg daily for three months	Follow up for recurrence	
Moderate lesions (5–10 cm) with bone involvement	Itraconazole 400 mg daily for six months	Wide local excision at six months	Itraconazole 400 mg daily for another six months	Follow up for recurrence
Massive lesions (>10 cm) with bone involvement and secondary bacterial infection	Itraconazole 400 mg daily for six months with repetitive lesion surgical debridement	Wide local excision at six months	Itraconazole 400 mg daily for another six months	Amputation for •Multiple surgical recurrences •No response to medical treatment •Life saving

## Anaesthesia

**Fig 2:**
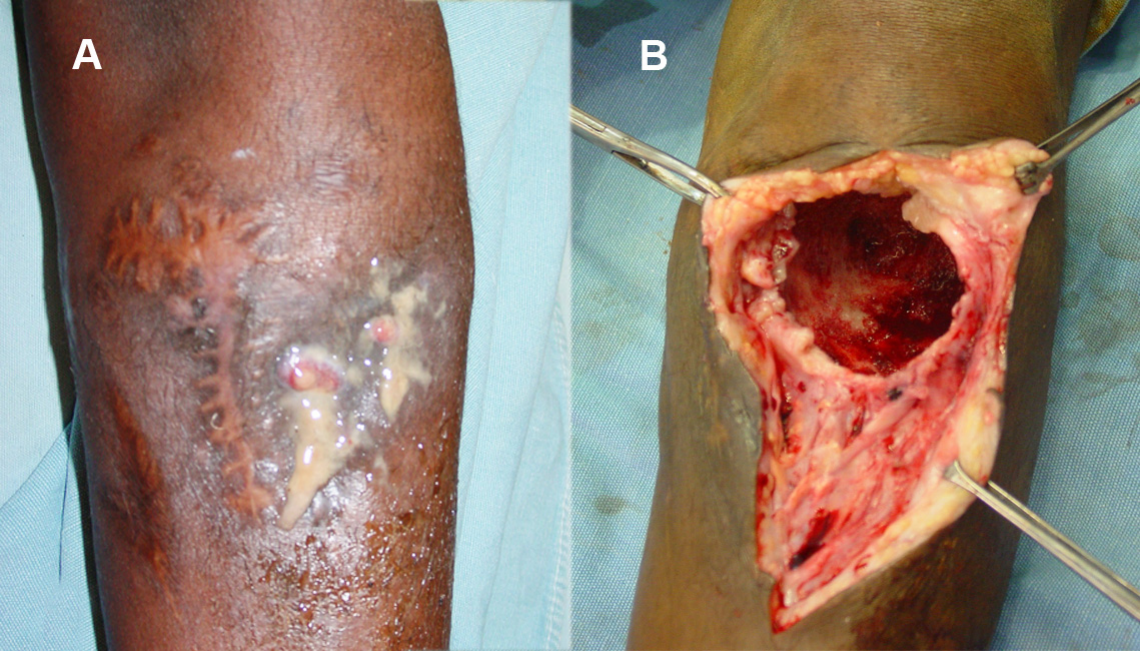
(A) Photograph of a patient with eumycetoma of the upper leg with minimal external appearance. (B) The same patient at surgical exploration, showing massive bone cavity.

In mycetoma, the spread of the disease along tissue planes is usually unpredictable, and the external appearance of the lesions is always deceiving, thus making complete surgical excision under local anaesthesia unfeasible; hence it is contraindicated in mycetoma (Fig 2A and 2B). Surgery is commonly performed under general anaesthesia, spinal analgesia, or block anaesthesia.

## Surgical Techniques

The surgical options for mycetoma treatment range from wide local excision to repetitive debridement excisions to amputations as well as various skin cover techniques.

A tourniquet-facilitated bloodless field is mandatory to identify the lesion margins in an attempt to avoid bursting it. This will reduce risks of disease local spread along tissue planes and future postoperative recurrences [30].

Generous long incisions in mycetoma are obligatory for adequate wide local surgical excisions. Ultrasound-guided surgical incisions can be helpful in accurately determining the site, size, and extent of the lesion [16].

Careful and meticulous surgical dissection with a good safety margin around the lesion is required for adequate wide local excision to reduce the incidence of postoperative recurrence. It is interesting to note that the nerves and tendons are rarely involved in mycetoma [4], and extra care must be observed in dissecting these structures to avoid postoperative neurological and locomotor complications.

**Fig 3:**
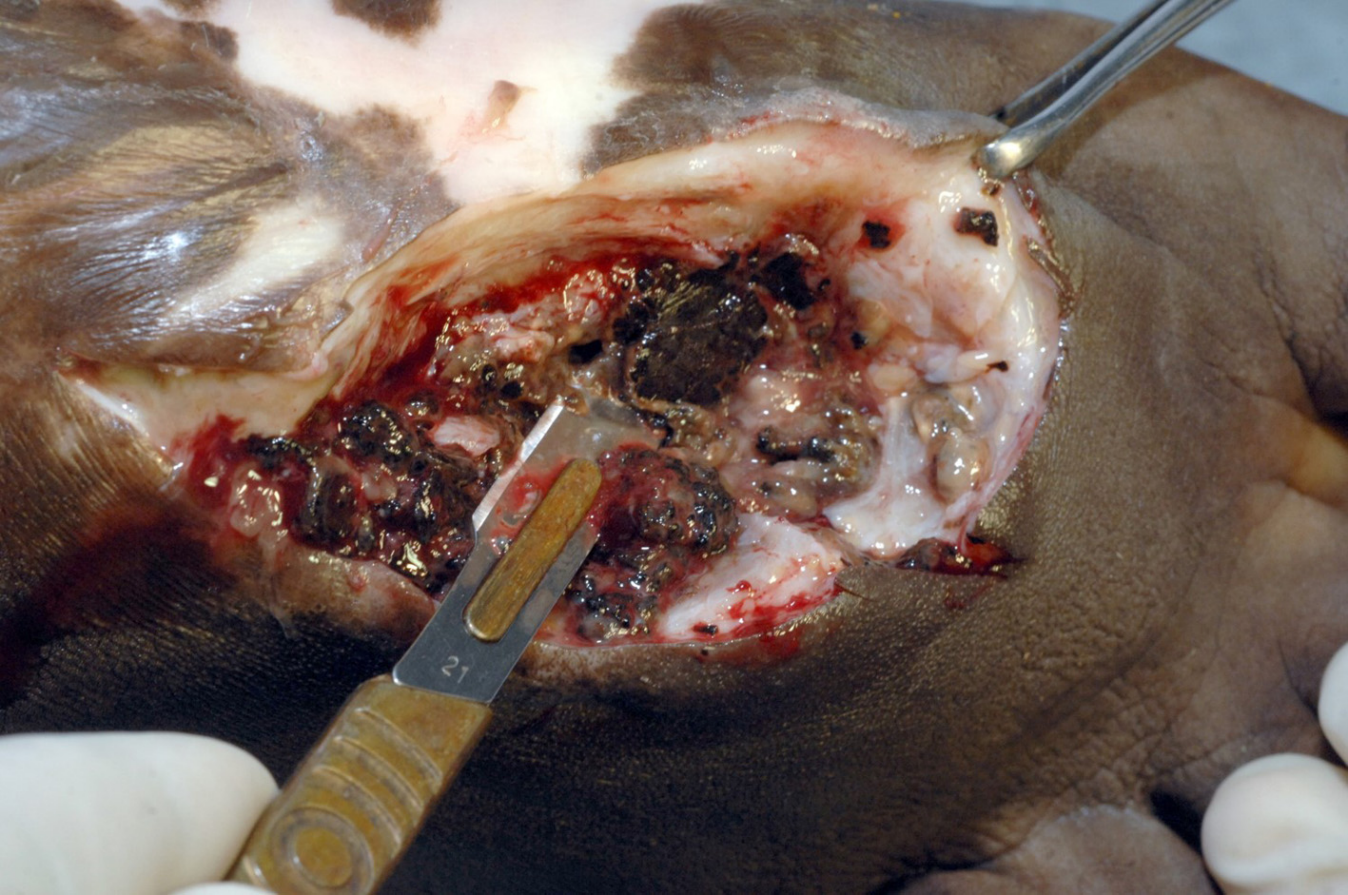
Photograph showing massive eumycetoma lesion. The bone is studded with multiple cavities cemented with massive grains and fibrous tissue.

**Fig 4:**
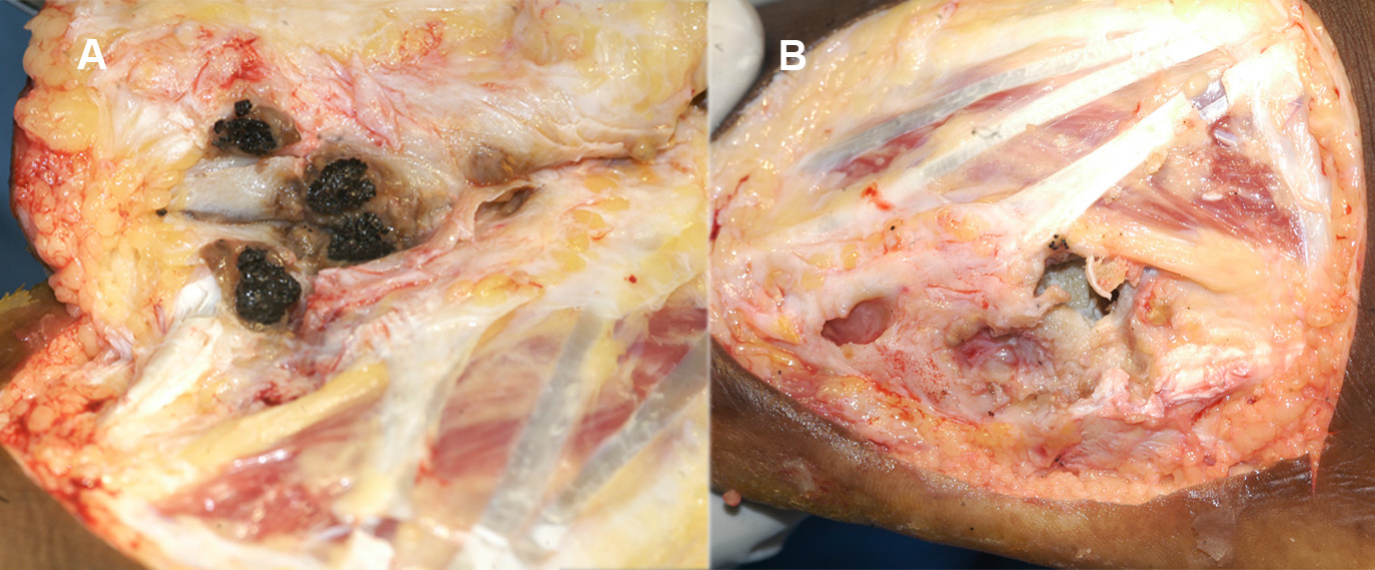
(A) Photograph showing multiple subcutaneous and bone cavities studded with grains at surgery. (B) The same cavities after surgical excisions.

In advanced disease, bone involvement is frequent (Figs 2B and 3). The bones are commonly studded with multiple cavities cemented with massive grains and fibrous tissue. Bone debridement, scooping the grains and fibrous tissue from the bone cavities, must be done meticulously (Figs 2B, 4A and 4B).

**Fig 5:**
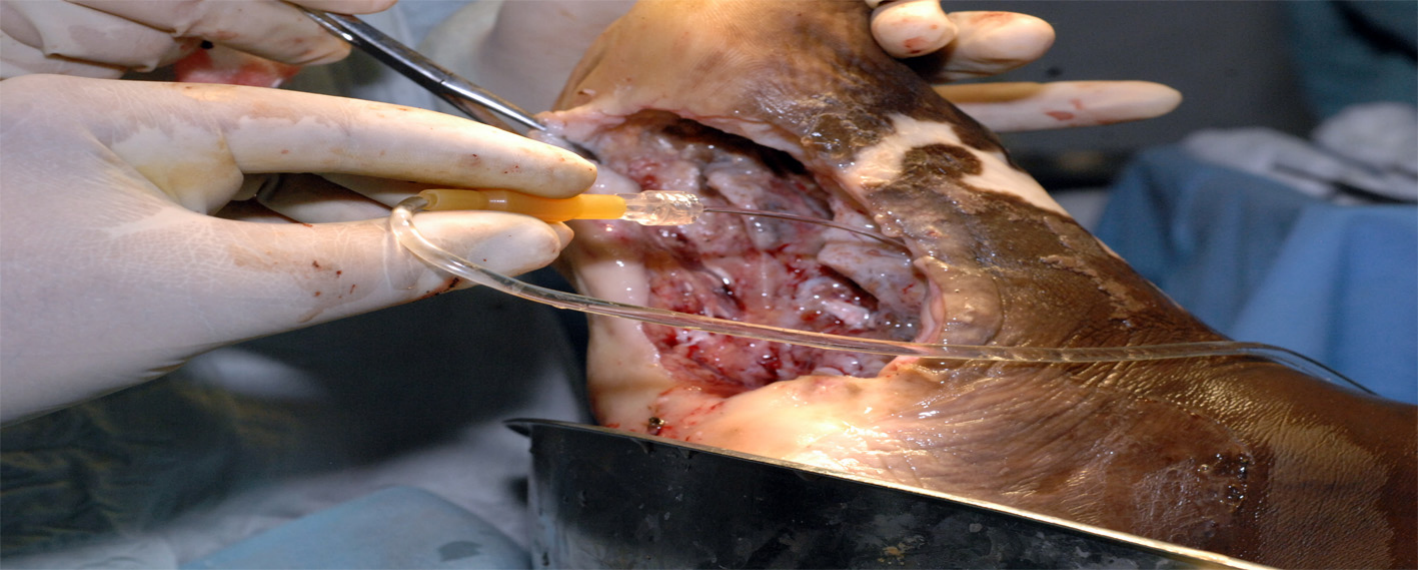
Photograph showing intraoperative wound irrigation with normal saline.

In mycetoma, the infection usually spreads widely along the tissue planes, forming deep-seated pockets that are not easily detected. These pockets must be identified carefully and precisely if postoperative recurrence is to be reduced. All damaged tissue must be debrided meticulously. Thorough and vigilant irrigation of the operative field with normal saline solution to remove any missed grains or infected tissue must be applied after debridement (Fig 5). Lastly, the surgical field must be rinsed and irrigated methodically with iodine solution and hydrogen peroxide solution several times to remove and destroy any missed grains and hyphae [31].

**Fig 6:**
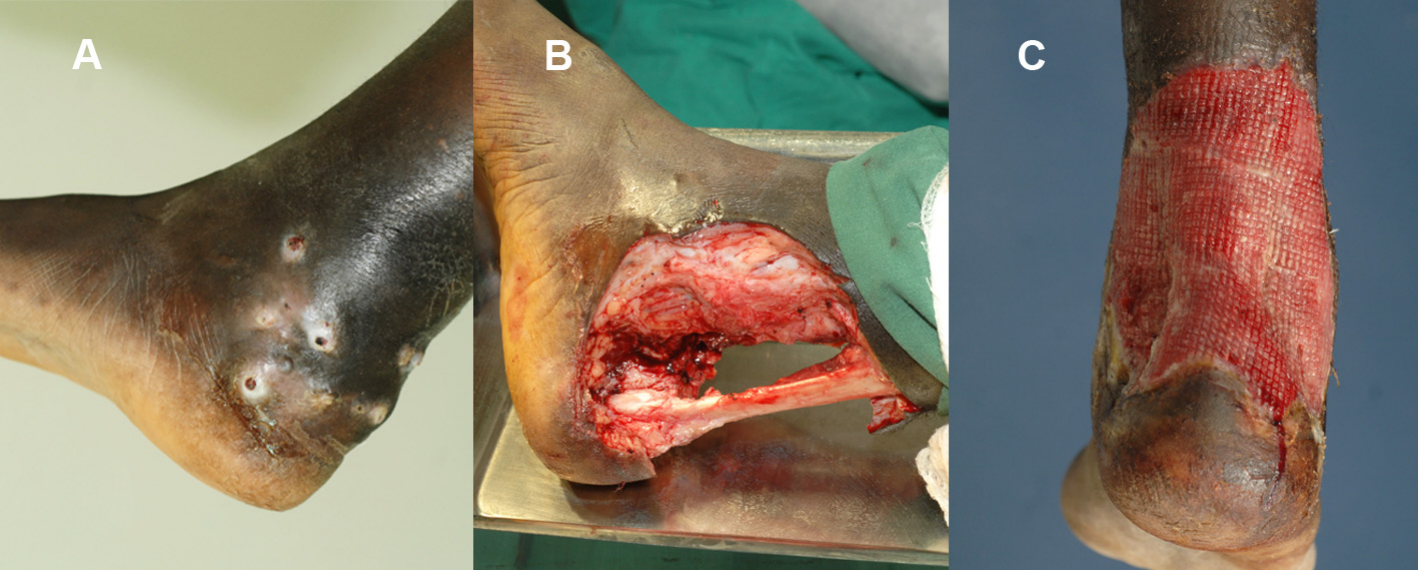
(A) Photograph showing an ankle-region eumycetoma. (B) The same lesion after wide local surgical excision. (C) The lesion with good granulation tissue.

The skin is closed primarily in small localised lesions following an adequate wide local excision provided that the skin is not involved and not under severe tension. Occasionally, skin undermining may be necessary to release skin tension. In large lesions, the wounds are allowed to heal with secondary intension and fibrous tissue or are closed by skin grafting at a later stage after the development of good granulation tissue (Fig 6A–6C).

It is interesting to note that the healing is adequate and fast in mycetoma, and this can be explained by the brisk blood supply to the mycetoma lesions as previously documented histopathologically and angiographically [32].

## Postoperative Care

Mycetoma patients require adequate postoperative analgesia as, frequently, the dissection and tissue resection is extensive. The peripheral circulation must be regularly monitored, and the wound dressing must be loosened postoperatively to avoid ischaemia.

Early mobilization and physiotherapy are mandatory for better surgical outcomes and to avoid the joint stiffness and reduce deformities and disabilities.

The opened surgical wounds require regular dressing, and there are different antiseptic solutions in use for this purpose. Based on literature reports and a recent study on the effects of different antiseptic solutions on *Madurella mycetomatis* isolates, hydrogen peroxide solution showed superiority in destroying this organism, and hence it is recommended as the antiseptic solution of choice for postoperative wounds dressing [31].

## Complications

Surgery for mycetoma has many early and late complications. The early and serious complication is ischaemia. Ischaemia occurs usually due to accidental vascular injury during surgical dissection, which is commonly seen in the digits. Alternatively, ischaemia can occur due to tight wound bandaging, which is applied to reduce the reactionary bleeding. Severe ischaemia frequently leads to gangrene, and mild ischaemia can cause skin necrosis and healing with hypopigmentation.

Intraoperative nerve injury may lead to paralysis and disability. All these complications can be reduced by meticulous surgical dissection.

Wound infection is a recognised postoperative complication, particularly in patients with preoperative secondary bacterial infection. To reduce this risk, it is essential to administer the appropriate prophylactic antibiotics at induction of anaesthesia and to continue postoperatively as necessary.

Late complications include deformities, disabilities, non-healing ulcers, and thick surgical scars, which are commonly due to healing by secondary intension and fibrosis. These can be reduced by wound skin grafting, meticulous physiotherapy, and using special shoes.

## The Postoperative Recurrence

The postoperative recurrence rate varies from 25%–50%. The postoperative recurrence can be local, distant at the regional lymph nodes, or further to distal organs such as the lung, abdominal cavity, or spinal cord [33–35]. This recurrence may be due to the disease biology and behaviour with inherent recurrence ability, inadequate surgical excision due to the use of local anaesthesia, lack of surgical experience, or drug compliance due to financial constraints and/or lack of health education.

Moreover, the organisms are usually embedded in the grains and cement material, which provide the organisms with more protection against body defence mechanisms and antifungal agents, thus contributing to postoperative recurrence [36].

It was observed in some histopathological sections that fungus hyphae were present free in different tissue plans, enabling them to spread widely and rendering them difficult to identify during surgery, leading to further reasons for recurrence.

## Hospital Cross Infection in Mycetoma

In many centres, mycetoma patients are placed at the end of the surgical list, on separate lists, or in septic surgical theatres. Commonly, the standard recommended time to achieve sterilization of the instruments is exceeded and the drapes and towels are destroyed for the fear of hospital cross infection. However, there is no evidence to support this practice, and the medical literature has not citied any reports of mycetoma due to hospital cross infection. Furthermore, the causative organisms are weak and can easily be eliminated by the standard sterilization techniques. In experimental animals, it was difficult to reproduce mycetoma granuloma by inoculation of the causative organisms; however, this was partially successful in immune-compromised mice and after the addition of an adjuvant to distract the host immune system in immune-competent mice [37].

**Fig 7:**
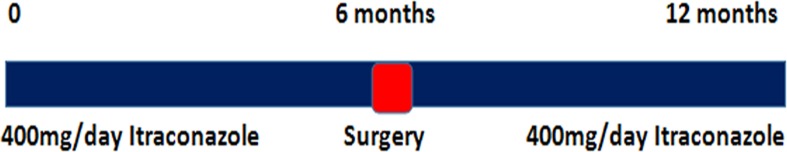
The mycetoma treatment protocol.

In conclusion, surgical intervention is an integral and rewarding component in the management of mycetoma (Fig 7). It facilitates the diagnosis and treatment of the disease. However, it should be performed in a suitably equipped surgical facility under experienced anaesthetic, surgical, and nursing personnel. The surgical procedure should be meticulously performed to reduce complications and achieve an optimal treatment outcome.

**Key Learning Points**

- Surgical intervention is an integral part of mycetoma management.
- Local anaesthesia is contraindicated in mycetoma surgery.
- Bloodless surgical fields and meticulous surgical techniques are essential for good treatment outcomes.
- Surgery should be conducted in a suitable, safe surgical environment to reduce its complications.
- There is no reported mycetoma case due to hospital cross infection.

**Top Five Papers**

1. Fahal AH, EL Hassan AM, Mahgoub ES, Rahman ME. (2015) Mycetoma in the Sudan: The Mycetoma Research Centre Update. PLoS Negl Trop Dis. 2015 Mar 27; 9(3):e0003679. doi: 10.1371/journal.pntd.0003679. eCollection 2015 Mar.
2. Fahal AH, Shaheen S, Jones DH (2014) The orthopaedic aspects of mycetoma. Bone Joint J 96-B: 420–425.
3. Zein HA, Fahal AH, Mahgoub el S, El Hassan TA, Abdel-Rahman ME (2012) Predictors of cure, amputation and follow-up dropout among patients with mycetoma seen at the Mycetoma Research Centre, University of Khartoum, Sudan. Trans R Soc Trop Med Hyg 106: 639–644.
4. Fahal AH. Mycetoma in Williams, Bulstrode, O'Connell. (2013) Bailey and Love's Short Practice of Surgery 26E: 26th Edition, Oxford University Press, 2013, pp 64–68.
5. Welsh O, Vera-Cabrera L, Salinas-Carmona MC. (2013) Current treatment for nocardia infections. Expert Opin Pharmacother. Dec;14(17):2387–98. doi: 10.1517/14656566.2013.842553.
